# Early functional recovery outcomes and return to work after primary total hip arthroplasty: *a novel patient reported outcomes questionnaire*

**DOI:** 10.1186/s13018-024-04937-z

**Published:** 2024-07-26

**Authors:** David Fawley, Thierry Bernard, Henry Clayton Thomason, Luigi Zagra, René H.M. ten Broeke, Kory Johnson

**Affiliations:** 1 DePuy Synthes, 700 Orthopaedic Drive, Warsaw, IN USA; 2 Carolina Orthopaedic & Sports Medicine Center, 2345 Court Dr, Gastonia, NC USA; 3 Hip Department, IRCCS Istituto Ortopedico Galeazzi, Milan, Italy; 4 Department of Orthopaedic Surgery, Maastricht University Medical Centre, Maastricht, 6202 AZ The Netherlands; 5 Orthopaedic Associates of Michigan, 555 Mid Towne St Suite 105, Grand Rapids, MI USA

**Keywords:** Total hip arthroplasty, Early recovery, Functional recovery outcomes, Return to work, Patient reported outcomes

## Abstract

**Purpose:**

Clinical and patient reported outcomes are often collected before and after the procedure to benchmark and study outcomes for patients. These outcomes and scores are useful for tracking patient outcomes after surgery, however, the fact that these commonly used measures typically provide information about a patient’s level of pain and function at a single point in time is a limitation.

**Methods:**

We present early functional recovery and return to work outcomes after primary THA from a novel questionnaire administered in a global, multi-center, prospective clinical study.

**Results:**

By 6 and 12 weeks post-op, a large proportion of study subjects were able to perform functional recovery outcomes after their THA: walk without an aid (74%; 94%); drive (76%; 97%); basic activities of daily living (94%; 99%); perform light household duties (91%; 96%); perform moderate-to-heavy household duties (54%; 86%); go up and down a flight of stairs (92%; 99%); put on socks/stockings (77%; 93%); bend down to pick up an object from the floor (87%; 97%); stand up from a chair (96%; 99%); perform leisure recreational activities (54%; 84%); perform primary goal identified pre-THA (69%; 86%). 60% were able to return to work by 12 weeks post-op. These questions showed strong association with the Forgotten Joint Score.

**Conclusion:**

Excellent patient reported early functional recovery outcomes and satisfaction were observed at 6- and 12-weeks post-op in this cohort and is the first reported data using a novel PRO.

**Clinical trial registration:**

NCT03189303, registered June 14, 2017.

**Supplementary Information:**

The online version contains supplementary material available at 10.1186/s13018-024-04937-z.

## Introduction

Total hip arthroplasty (THA) has long been viewed as one of the most successful surgical procedures in the world [1]. However, there is increasing interest in recovery during the early post-operative period to help provided distinction between treatment options. One major area of interest has been surgical approach; many studies have reviewed the effect of minimally invasive approaches, including the direct anterior approach, on early outcomes [2–5]. Studies have also been conducted to help compare early recovery for different implant designs [6], recovery pathways [7–9], and technologies such as robotics and navigation [10].

Clinical and patient reported outcomes (PROs) are often collected before and after the procedure to benchmark and study outcomes for patients. These outcomes and scores are useful for tracking patient outcomes after surgery, however, the fact that these commonly used measures typically provide information about a patient’s level of pain and function at a single point in time is a limitation. Some have used traditional outcomes scores, administered each week after surgery to study early recovery [5, 11]. We have developed a questionnaire that allows collection of these early functional recovery data out to 12 weeks post-surgery with a goal of offering a solution with less burden for the patient and provider. The questions we have developed are like those that one would find on a traditional PRO, but we have modified the application. We developed a PRO that allows the patient to record the week when they could first perform an activity after their surgery. This allows us to review the course of recovery over time and not just recovery at a single post-operative timepoint (i.e., 6-weeks). The purpose of this study was: (1) determine how quickly patients could perform functional recovery activities between week 1 and week 12 postoperatively, including return to work after their surgery, and (2) review any potential associations between these questions and the Forgotten Joint Score (FJS-12).

## Materials and methods

A global, prospective, multi-center study was conducted between August 2017 and May 2021. Study sites participated from the United States, The Netherlands, Wales, Scotland, and Italy. Informed consent was collected for all subjects prior to participation and Institutional Review Board (IRB), or Ethics Committee (EC) review and approval was obtained for the study protocol and all research sites and maintained through the duration of the study. Patients were excluded if they were being treated for a muscular disorder that limited mobility, if they were bedridden, if they were pregnant or lactating, if they had previous partial or total hip replacement on the study hip, active infection (local or systemic), and if the contralateral hip had been replaced less than 6 months prior. Preoperative assessments included Harris Hip Score [12], EQ-5D-5 L [13, 14], and baseline patient reported early functional recovery outcomes and pain. A PINNACLE™ acetabular cup (DePuy Synthes, Warsaw, IN) was used in all cases with a Corail™, ACTIS™ or SUMMIT™ stem (DePuy Synthes, Warsaw, IN). Surgeries were performed via posterolateral, anterolateral, or lateral approach with the subject in lateral decubitus position. No imaging or navigation was used to aid in placement of the acetabular components. Date of discharge and discharge disposition was recorded. Postoperative assessments included Harris Hip Score, EQ-5D-5 L, FJS-12 [15], and patient reported early functional recovery outcomes and pain. Complications (all serious and device- or procedure-related) were recorded from the time of subject consent to end of study participation.

### Development of the questionnaire

The functional recovery outcomes questionnaire (Appendix A) includes both a preoperative and a postoperative form. The preoperative form is used to establish a baseline. The subject is asked if they can perform a specific activity with available responses of “Yes”, “No, because of study hip”, and “No, for reason other than study hip”. It is important to understand whether the subject can perform the activity in question before their surgery, or whether their health or other factors are preventing their participation in these activities. On the postoperative form, the subject is asked to note within which week after the surgery they were first able to perform the activity, up to 12 weeks postoperatively, or to report “>12 weeks” or “still cannot do”. A response of “N/A” was expected if the subject stated preoperatively that they were not able to perform a specific task or activity for a reason other than their study hip. A return-to-work question was also included to assess when the subject was able to return to work after surgery, and their work status, full- or part-time. Patients completed the questionnaire at their 6-week and 12-week visits. In cases where the patient could complete all activities at the 6-week visit, they did not have to fill out the form again when they returned at 12 weeks. In other words, once completion of an activity was documented post-operatively, the patient was not asked again to address this at a subsequent visit. In the cases where subjects did not speak English, translations of the questions were provided.

### Statistical analysis

Clinical assessments were summarized with sample size, mean, and standard deviation for numeric scores and with sample size and percentages for categorical response using J&J’s corporate SAS server, version 9.4. Changes from baseline were tested for significance using the 2-sided paired t-test. Associations between each question and the FSJ-12 were explored using a t-test.

## Results

### Patient demographics

**Fig. 1:**
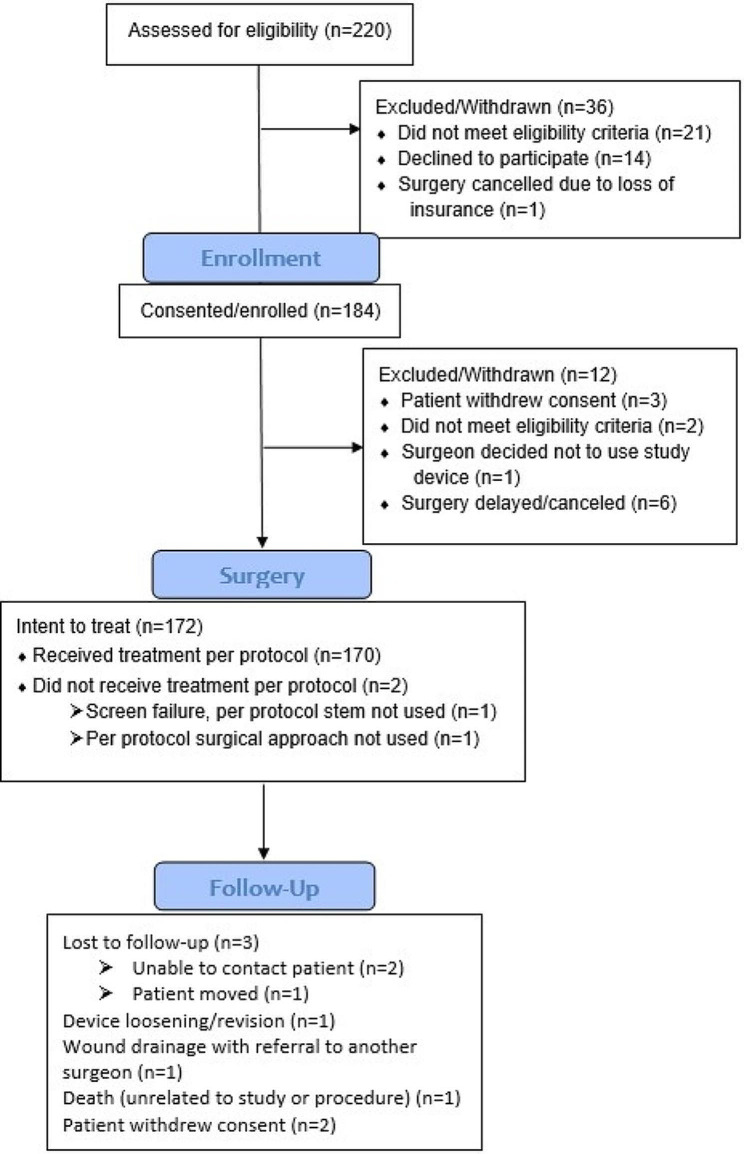
Patient enrollment flowchart

184 patients were enrolled in the study. A total of 170 subjects were enrolled and treated per the study protocol (Fig. 1). Mean age at surgery was 65.8 (range 35 to 85). Gender was female in 63.5% of cases and primary diagnosis was osteoarthritis for 88.2% of cases. Mean BMI was 29.7 (range 17.4 to 54.6). Additional demographic and surgical details are presented in Table 1.

**Table 1:** Patient demographics and surgical details

	Mean	SD	Range	n	
**Age (years)**	**65.8**	**8.9**	**35 to 85**	**170**	
**BMI (kg/m** ^**2**^ **)**	**29.7**	**6.1**	**17.4 to 54.6**	**170**	
**Skin-to-skin time (minutes)**	**62.0**	**18.0**	**33 to 119**	**170**	
**Incision measurement (cm)**	**14.6**	**3.0**	**8 to 21**	**167**	
**Length of stay (days)**	**2.1**	**1.6**	**0 to 7**	**170**	
**Gender**	**Female** 108 (63.5%)	**Male** 62 (36.5%)			
**ASA Risk**	**I** 17 (10.0%)	**II** 113 (66.5%)	**III** 39 (22.9%)	**IV** 1 (0.6%)	
**Primary Diagnosis**	**OA** 150 (88.2%)	**AVN** 8 (4.7%)	**CDH/DDH** 7 (4.1%)	**Other** 5 (2.9%)	
**Operative Side**	**Left** 79 (46.5%)	**Right** 91 (53.5%)			
**Bone Class**	**Normal** 75 (44.1%)	**Good** 77 (45.3%)	**Fair** 9 (5.3%)	**Poor** 2 (1.2%)	**Sclerotic** 7 (4.1%)
**Surgical Approach**	**Posterior** 108 (63.5%)		**Anterolateral (Modified Hardinge)** 62 (36.5%)		
**Discharge Disposition**	**Home** 115 (67.6%)	**Home Health Care** 37 (21.8%)		**Short-term Rehab Facility** 18 (10.6%)	

Mean Harris Hip Scores (SD; N) were 51.3 (17.1; 141), 84.2 (14.2; 124), and 92.7 (9.7; 143) at pre-op, 6- and 12-weeks post-op respectively. Mean EQ-5D-5 L scores (SD; N) were 0.59 (0.18; 169), 0.79 (0.13; 151), and 0.85 (0.14; 157) at pre-op, 6- and 12-weeks post-op respectively. Mean EQ-5D-5 L VAS were 67.9 (19.1; 169), 80.9 (14.1; 151), and 83.6 (13.6; 158) at pre-op, 6- and 12-weeks post-op respectively. Subjects reported being extremely or very satisfied post-operatively at a rate of 93.2%. Additional details are presented in Table 2.

**Table 2:** Clinical outcomes

		Mean	SD	*n*	*p*-value^§^
**Harris Hip Total Score (range 0-100)**					
Pre-operative 6-week (14–60 days)^Ω^ 12-week (61–180 days)		**51.3** **84.2** **92.7**	**17.1** **14.2** **9.7**	**141** **124** **143**	< **0.0001** < **0.0001**
**EQ-5D-5 L**					
Pre-operative		**0.59**	**0.18**	**169**	
6-week (14–60 days) 12-week (61–180 days)		**0.79** **0.85**	**0.13** **0.14**	**151** **157**	< **0.0001** < **0.0001**
**EQ-5D VAS (range 0-100)**					
Pre-operative		**67.9**	**19.1**	**169**	
6-week (14–60 days) 12-week (61–180 days)		**80.9** **83.6**	**14.1** **13.6**	**151** **158**	**< 0.0001** **< 0.0001**
**Forgotten Joint Score (range 0-100)**					
Pre-operative		**-**	**-**	**-**	
6-week (14–60 days)		**46.0**	**29.0**	**151**	
12-week (61–180 days)		**63.4**	**28.3**	**158**	**< 0.0001**
		**None**	**Mild**	**Moderate**	**Severe**
**Buttock Pain (n (%))**					
Pre-operative 6-week (14–60 days) 12-week (61–180 days)		**34 (20.1)** **87 (51.2)** **103 (60.6)**	**42 (24.9)** **45 (26.5)** **41 (24.1)**	**65 (38.5)** **17 (10.0)** **13 (7.6)**	**28 (16.6)** **2 (1.2)** **0 (0.0)**
**Groin Pain (n (%))**					
Pre-operative 6-week (14–60 days) 12-week (61–180 days)		**23 (13.6)** **77 (45.3)** **110 (64.7)**	**21 (12.4)** **50 (29.4)** **36 (21.2)**	**78 (46.2)** **20 (11.8)** **10 (5.9)**	**47 (27.8)** **4 (2.4)** **2 (1.2)**
	**Extremely satisfied**	**Very satisfied**	**Moderately satisfied**	**Slightly satisfied**	**Not at all satisfied**
**Patient Satisfaction (n (%))**					
Pre-operative* 6-week (14–60 days) 12-week (61–180 days)	**64 (37.6)** **82 (48.2)** **93 (54.7)**	**95 (55.9)** **60 (35.3)** **53 (31.2)**	**7 (4.1)** **8 (4.7)** **11 (6.5)**	**0 (0.0)** **1 (0.6)** **1 (0.6)**	**3 (1.8)** **0 (0.0)** **0 (0.0)**
			**Equal**	**Right longer**	**Left longer**
**Patient Perception of Leg Length (n (%))**					
Pre-operative 6-week (14–60 days) 12-week (61–180 days)			**118 (71.5)** **24 (14.5)** **23 (13.9)**	**115 (67.6)** **19 (11.2)** **12 (7.1)**	**130 (76.5)** **13 (7.6)** **11 (6.5)**

^§^change from baseline

^Ω^ROM was not collected at the 6-week visit for some subjects, per surgeon discretion

*pre-op is patient expectation of satisfaction

**Fig. 2:**
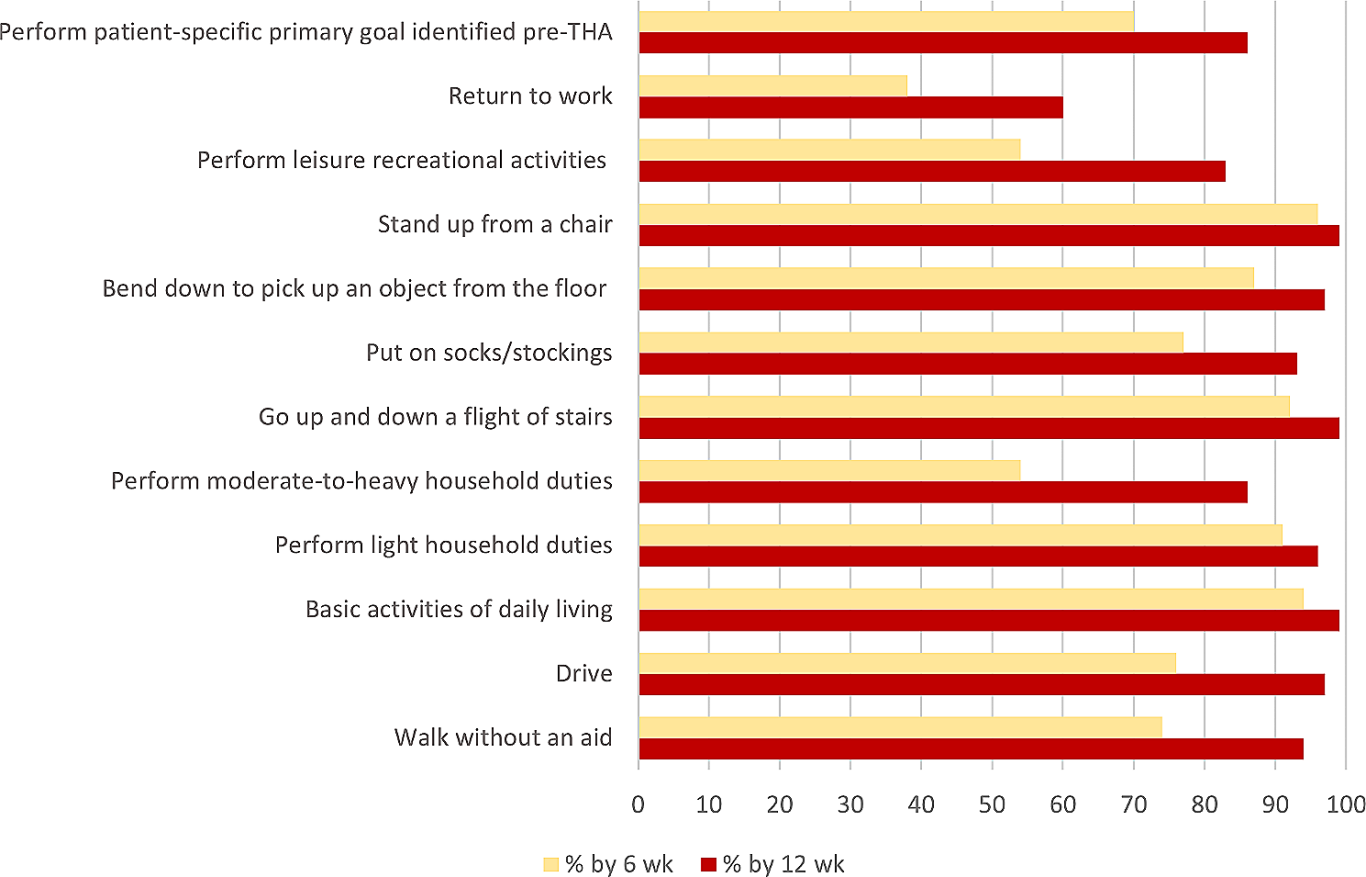
Functional Recovery Outcomes (% subjects who responded that they could perform activity at 6- and 12-weeks postoperatively)

**Fig. 3:**
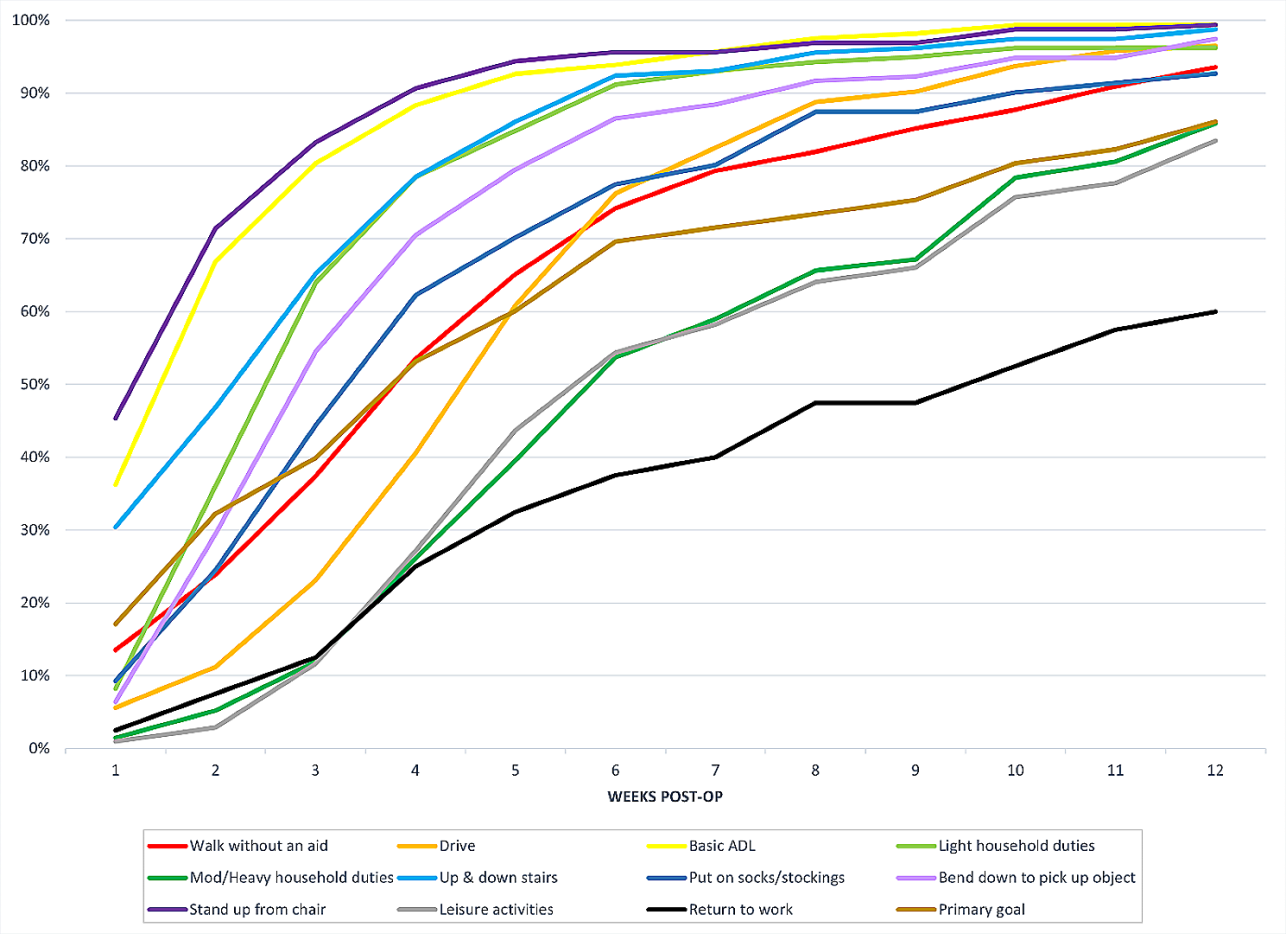
Functional recovery outcomes over time

The following patient reported functional recovery outcomes (Fig. 2) will be presented as the percentage of subjects that reported they could first complete the activity without assistance at or before 6 and 12 weeks post-op (6wk; 12wk): walk without an aid (74%; 94%); drive (76%; 97%); basic activities of daily living (94%; 99%); perform light household duties (91%; 96%); perform moderate-to-heavy household duties (54%; 86%); go up and down a flight of stairs (92%; 99%); put on socks/stockings (77%; 93%); bend down to pick up an object from the floor (87%; 97%); stand up from a chair (96%; 99%); perform leisure recreational activities (54%; 84%); perform primary goal identified pre-THA (69%; 86%). Figure 3 shows the cumulative response rate over time for patient reported functional recovery outcomes. As expected, there was a difference between sides for time to drive after THA. By week 5 post-op, the percentage who could drive was similar (62% for right THA; 60% for left THA) (Table 3).

**Table 3:** Time to first drive by side

	Wk 1	Wk 2	Wk 3	Wk 4	Wk 5	Wk 6	Wk 7	Wk 8	Wk 9	Wk 10	Wk 11	Wk 12
Right THA (*n* = 76)	1%	8%	20%	38%	62%	71%	82%	88%	89%	93%	95%	96%
Left THA (*n* = 67)	10%	15%	27%	43%	60%	82%	84%	90%	91%	94%	97%	97%

For applicable subjects (those working before surgery and those that did not select “Not Applicable” post-surgery), 38% had returned to work by 6 weeks, and 60% had returned by 12-weeks post-operatively. No significant association was found between early recoveries and age, sex, BMI, and ASA risk.

A strong association between early recovery and FJS-12 scores was found (Table 4). The only questions that did not show some level of significant association were performance of light household duties, putting on socks/stockings without assistance, and bending down to pick up an object; this was likely due to most patients having recovered by 6 weeks for these, though for all questions, the mean FJS-12 was higher for those patients that recovered by or before 6 weeks. Recoveries between surgical approaches were reviewed but no significant differences were noted between the two approaches.

**Table 4:** Associations with FJS-12

Question		Recovery at or before 6 weeks	No recovery or recovery after 6 weeks	T test *p*-value
First walk without an aid after surgery	n	104	38	**0.0028**
	**Mean FJS-12**	**50.6**	**34.1**	
First drive after surgery	n	99	32	**0.0019**
	**Mean FJS-12**	**50.5**	**32.7**	
First perform basic activities without assistance	n	142	8	**0.0056**
	**Mean FJS-12**	**47.4**	**18.4**	
First perform light household duties	n	133	12	0.1175
	**Mean FJS-12**	**46.9**	**33.2**	
First perform moderate/heavy household duties	n	65	56	**0.0299**
	**Mean FJS-12**	**53.5**	**42.1**	
First Go up and down a flight of stairs	n	137	9	**< 0.0001**
	**Mean FJS-12**	**48.5**	**12.7**	
First put on socks/stockings without assistance	n	108	31	**0.0003**
	**Mean FJS-12**	**50.6**	**29.5**	
First bend down to pick up an object	n	127	16	0.4200
	**Mean FJS-12**	**46.8**	**40.5**	
First stand up from a chair without assistance	n	144	4	0.1257
	**Mean FJS-12**	**46.4**	**23.8**	
First participate in leisure activities	n	52	40	**0.0013**
	**Mean FJS-12**	**54.4**	**35.7**	
First return to work after surgery	n	14	21	**0.0399**
	**Mean FJS-12**	**56.0**	**34.4**	
First able to accomplish the primary goal identified	n	101	45	**0.0006**
	**Mean FJS-12**	**51.4**	**33.7**	

## Discussion

To our knowledge, our functional recovery outcomes questionnaire (FROQ) is the first questionnaire of its kind that allows collection of early functional recovery data out to 12 weeks post-THA surgery with a goal of offering a solution with less burden (patient visits) for the patient and provider. The data collection allows a more granular view of patient recovery (weekly) without requiring patients to keep a diary or add follow up visits. Our goal in introducing this questionnaire was to allow providers to gather recovery data during the early post-operative period to help compare implants, surgical approaches, recovery pathways, and technologies to optimize patient outcomes.

It is well established that preoperative function and pain are predictors of postoperative outcomes, especially in the short-term follow up period [16–19]. Emotional state [20, 21], mental health status [22, 23], less post-operative restrictions [24], and higher levels of activity [25] have also been correlated with better early postoperative function. Gender has also been linked to differing outcomes [26]. Average RTW after THA varies in the literature, ranging between 1.1 and 13.9 weeks [27–34]. A systematic review and meta-analysis conducted by Hoorntje et al. [28] showed a mean RTW time of 8.9 weeks, and RTW rates as high as 86% for those who were working preoperatively. In our cohort, 85% of subjects returned to work post-surgery, for all subjects who worked full or part time prior to surgery or could not work due to their study hip. Of the 34 subjects who returned to work, 50% returned to work at a mean of 7.7 weeks.

Rolfson et al. [35] reported that pre-operative anxiety and depression (measured using the EQ-5D dimension of anxiety/depression) was a strong predictor of pain relief and satisfaction after surgery. In future evaluations it could be of interest to review compared to the FROQ. The relationship between pain and anxiety and depression is complex. To better understand the relationship, the factors for each patient’s pain and recovery pathway need to be identified.

Porsius et al. [11] reviewed recovery in fast-track primary THA compared with patient characteristics. In this analysis they conducted latent class growth modelling (LCGM) to improve insight into heterogenous data. The outcome measure used was the Oxford Hip Score (OHS) and was completed by subjects 1 week preoperatively and weekly for 6 weeks after surgery. In this study the authors use a well-known outcome measure (OHS) but burden is added for subjects who are required to fill out the entire form a total of seven times up to 6 weeks post-surgery. This approach is feasible only in limited research applications, but the functional recovery outcomes questions that we used for this study allow the subject to complete the questionnaire at their standard-of-care office visits.

We acknowledge that our study is not without any limitations. Limitations of the study include a lack of performance-based functional measures since it has been shown that patients tend to overestimate their functional recovery after THA [36]. It would also have been beneficial to measure psychological factors preoperatively since patients with low levels of depression and anxiety are more likely to have better early functional outcomes [22]. It would have been beneficial to know analgesia information for patients post-operatively as this is an important factor to consider for functional recovery. Potential recall bias is another limitation. Since the form was administered at the 6- and 12-week visits, there is potential that patients had difficulty accurately recalling when they were able to complete functional activities post-surgery. Another limitation of this study is a lack of comparative cohort or control group. There is also inherent difficulty in collecting detailed baseline status for our functional recovery questions. For instance, use of an aid varies widely between patients (full assistance with a walker versus occasional use of a cane); however, this is a difficulty for all patient reported questionnaires as there are limitations on the number and complexity of questions a patient can be expected to answer, particularly in a standard of care setting. There was a strong association between early recovery and higher FJS-12 scores. Further validation work would be beneficial to officially validate these early recovery questions, however, it is the intention of the authors to present these data to set a baseline for future studies and to add to the growing body of literature exploring ways to better understand and study early postoperative recovery after THA.

## Conclusion

Excellent patient reported early functional recovery outcomes and satisfaction were observed at 6- and 12-weeks post-op in this cohort and is the first reported data using a novel PRO. These early recovery outcomes questions associated strongly with the Forgotten Joint Score. These data could be beneficial for informing patients of potential functional recovery timelines and could be of greater interest, compared with PRO scores, in the early post-operative period.

### Electronic supplementary material

Below is the link to the electronic supplementary material.

Supplementary Material 1

## Data Availability

Data can be requested through the Yale Open Data Access (YODA) project: https://dev-yoda.pantheonsite.io/jj-available-data/.
