# Supplementary material for: Early functional recovery outcomes and return to work after primary total hip arthroplasty: a novel patient reported outcomes questionnaire
Source: J Orthop Surg Res. 2024 Jul 26;19:434. doi: 10.1186/s13018-024-04937-z (PMC11282613; doi:10.1186/s13018-024-04937-z)
Supplement: Supplementary file 1 — Supplementary Material 1 [file 13018_2024_4937_MOESM1_ESM.pdf]

Appendix A – Functional Recovery Outcomes Questionnaire

Pre-operative Questions

1. How satisfied do you anticipate you will be with this procedure?
☐ Extremely Satisfied ☐ Very satisfied ☐ Moderately satisfied ☐ Slightly satisfied ☐ Not at all satisfied

2. Groin pain: (Select one)
☐ None ☐ Mild ☐ Moderate ☐ Severe

3. Buttock Pain: (Select one)
☐ None ☐ Mild ☐ Moderate ☐ Severe

4. Leg Length:
☐ Equal ☐ Right side longer ☐ Left side longer

5. Can you walk without an aid?
☐ Yes ☐ No - because of study hip ☐ No - for reason other than study hip

6. Do you drive?
☐ Yes ☐ No - because of study hip ☐ No -for reason other than study hip

7. Can you perform basic activities of daily living without assistance (bathing, getting in and out of bed, etc.)?
☐ Yes ☐ No - because of study hip ☐ No - for reason other than study hip

8. Can you perform light household duties (cooking, dusting, etc.)?
☐ Yes ☐ No - because of study hip ☐ No - for reason other than study hip

9. Can you perform moderate to heavy household duties (cleaning floors, moving heavy boxes, etc.)?
☐ Yes ☐ No - because of study hip ☐ No - for reason other than study hip

10. Can you go up and down a flight of stairs using a handrail?
☐ Yes ☐ No - because of study hip ☐ No - for reason other than study hip

11. Can you put on socks/stockings without assistance?
☐ Yes ☐ No - because of study hip ☐ No - for reason other than study hip

12. Can you bend down to pick up an object on the floor?
☐ Yes ☐ No - because of study hip ☐ No - for reason other than study hip

13. Can you stand up from a chair without assistance?
☐ Yes ☐ No - because of study hip ☐ No - for reason other than study hip

14. Can you participate in leisure recreational activities (swimming, gardening, bowling, etc.)?
☐ Yes ☐ No - because of study hip ☐ No - because of reason other than study hip

15. Do you work?
☐ Yes, full time ☐ Yes, part time because of study hip ☐ No, because of study hip ☐ No, retired or for reason other than study hip

16. What is the primary goal you would like to accomplish after your hip surgery?

Post-operative Questions

1. How satisfied are you with this procedure?
☐ Extremely Satisfied ☐ Very satisfied ☐ Moderately satisfied ☐ Slightly satisfied ☐ Not at all satisfied

2. Would you have this procedure again?
☐ Yes ☐ No

3. Groin pain:
☐ None ☐ Mild ☐ Moderate ☐ Severe

4. Buttock Pain:
☐ None ☐ Mild ☐ Moderate ☐ Severe

5. Leg Length:
☐ Equal ☐ Right side longer ☐ Left side longer

6. When could you first walk without an aid after surgery to replace your study hip?
☐ Week 1 ☐ Week 2 ☐ Week 3 ☐ Week 4 ☐ Week 5 ☐ Week 6 ☐ Week 7 ☐ Week 8
☐ Week 9 ☐ Week 10 ☐ Week 11 ☐ Week 12 ☐ After Week 12 ☐ Not applicable ☐ Still cannot do

7. When could you first drive after surgery to replace your study hip?
☐ Week 1 ☐ Week 2 ☐ Week 3 ☐ Week 4 ☐ Week 5 ☐ Week 6 ☐ Week 7 ☐ Week 8
☐ Week 9 ☐ Week 10 ☐ Week 11 ☐ Week 12 ☐ After Week 12 ☐ Not Applicable ☐ Still cannot do

8. When could you first perform basic activities of daily living without assistance after surgery to replace your study hip (bathing, getting in and out of bed, etc.)?
☐ Week 1 ☐ Week 2 ☐ Week 3 ☐ Week 4 ☐ Week 5 ☐ Week 6 ☐ Week 7 ☐ Week 8
☐ Week 9 ☐ Week 10 ☐ Week 11 ☐ Week 12 ☐ After Week 12 ☐ Not Applicable ☐ Still cannot do

9. When could you first perform light household duties after surgery to replace your study hip (cooking, dusting, etc.)?
☐ Week 1 ☐ Week 2 ☐ Week 3 ☐ Week 4 ☐ Week 5 ☐ Week 6 ☐ Week 7 ☐ Week 8
☐ Week 9 ☐ Week 10 ☐ Week 11 ☐ Week 12 ☐ After Week 12 ☐ Not Applicable ☐ Still cannot do

10. When could you first perform moderate/heavy household duties after surgery to replace your study hip (cleaning floors, moving heavy boxes, etc.)?
☐ Week 1 ☐ Week 2 ☐ Week 3 ☐ Week 4 ☐ Week 5 ☐ Week 6 ☐ Week 7 ☐ Week 8
☐ Week 9 ☐ Week 10 ☐ Week 11 ☐ Week 12 ☐ After Week 12 ☐ Not Applicable ☐ Still cannot do

11. When could you first go up and down a flight of stairs using a handrail after surgery to replace your study hip?
☐ Week 1 ☐ Week 2 ☐ Week 3 ☐ Week 4 ☐ Week 5 ☐ Week 6 ☐ Week 7 ☐ Week 8
☐ Week 9 ☐ Week 10 ☐ Week 11 ☐ Week 12 ☐ After Week 12 ☐ Not Applicable ☐ Still cannot do

12. When could you first put on socks/stockings without assistance after surgery to replace your study hip?
☐ Week 1 ☐ Week 2 ☐ Week 3 ☐ Week 4 ☐ Week 5 ☐ Week 6 ☐ Week 7 ☐ Week 8
☐ Week 9 ☐ Week 10 ☐ Week 11 ☐ Week 12 ☐ After Week 12 ☐ Not Applicable ☐ Still cannot do

13. When could you first bend down to pick up an object on the floor after surgery to replace your study hip?
☐ Week 1 ☐ Week 2 ☐ Week 3 ☐ Week 4 ☐ Week 5 ☐ Week 6 ☐ Week 7 ☐ Week 8
☐ Week 9 ☐ Week 10 ☐ Week 11 ☐ Week 12 ☐ After Week 12 ☐ Not Applicable ☐ Still cannot do

14. When could you first stand up from a chair without assistance after surgery to replace your study hip?
☐ Week 1 ☐ Week 2 ☐ Week 3 ☐ Week 4 ☐ Week 5 ☐ Week 6 ☐ Week 7 ☐ Week 8
☐ Week 9 ☐ Week 10 ☐ Week 11 ☐ Week 12 ☐ After Week 12 ☐ Not Applicable ☐ Still cannot do

15. When could you first participate in leisure recreational activities (swimming, gardening, bowling, etc.) after surgery to replace your study hip?
☐ Week 1 ☐ Week 2 ☐ Week 3 ☐ Week 4 ☐ Week 5 ☐ Week 6 ☐ Week 7 ☐ Week 8
☐ Week 9 ☐ Week 10 ☐ Week 11 ☐ Week 12 ☐ After Week 12 ☐ Not Applicable ☐ Still cannot do

16. When did you first return to work after surgery to replace your study hip?
☐ Week 1 ☐ Week 2 ☐ Week 3 ☐ Week 4 ☐ Week 5 ☐ Week 6 ☐ Week 7 ☐ Week 8
☐ Week 9 ☐ Week 10 ☐ Week 11 ☐ Week 12 ☐ After Week 12 ☐ Not Applicable ☐ Still have not returned to work
If you did return to work, did you return full time or part time?
☐ Full time ☐ Part time

17. When were you first able to accomplish the primary goal you identified after surgery to replace your study hip?
☐ Week 1 ☐ Week 2 ☐ Week 3 ☐ Week 4 ☐ Week 5 ☐ Week 6 ☐ Week 7 ☐ Week 8
☐ Week 9 ☐ Week 10 ☐ Week 11 ☐ Week 12 ☐ After Week 12 ☐ Still cannot do
